# Homœopathy

**Published:** 1846-12

**Authors:** 


2. *Homœopathy.*—Dr. Linton thus discourses of Homœopathy; we extract from an article on that subject:

We assert that Homœopathy, whatever of truth there may be in some of its speculations, is perfectly inert in practice; and if we fail in proving this assertion true, then facts are mere illusions; logic a humbug, and reasoning a farce. To proceed. In the first place, our disposition to try all things, has induced us to try "*Globules.*" We have used them to ascertain their *pathogenetic* effects; we have taken the sulphur, but it caused nothing like the itch, which was promised us; we have used the quinine without experiencing the slightest symptoms of a chill; the belladonna, and nothing like hydrophobia followed. This we did at the suggestion of a Homœopathic practitioner. We have also tried these articles on some friends, without the slightest result. We have used the "globules" in affections which we were confident would *get well of themselves.* Here they were successful, the patient got well! But then we tried another experiment. We selected several cases which we felt confident would *not get well of themselves*, and these we subjected to the treatment of one who ranked high as a Homœopathic practitioner. The result was in every instance a complete and *triumphant failure.*

The following gives the author's opinion of the shaking, rubbing, and spiritualizing process:

But again say the defenders of small globules, *the rubbing—the trituration* of the medicines increases their power and activity. Some of them say that it spiritualizes matter to rub it! Hence they grind their medicines very fine, and shake the vial of drops—they rub about six minutes at each trituration, and shake about six times at each dilution, though Hahnemann says that he had to reduce his shakes, so powerful did six make it!!!!!

Now, any one that is in danger of believing this monstrous nonsense, can easily test its truth or falsehood. A certain amount of arsenic will kill a dog—a small dose, say half a grain, will not hurt him. Give the dog then a half grain of arsenic, and watch its effects. Then take another half grain and triturate, and grind it, and rub it, until it is *spiritualized* and *strengthened* as much as it is possible by this process. Then dilute it, and shake it well *sixty times six*, and give to the aforesaid dog. If Homœopathy be true, it will kill him in a very short time; if Homœopathy be false, the dog will go about his business. An easier test would be to ascertain if shaking a teaspoonful of brandy would enable it to make a man drunk. It would do so if Homœopathy be true.

Why, if this principle were sound, then the apothecary might double his stock at an hour's warning, not by the difficult and expensive process of importing fresh medicines, but by the easy one of *shaking* what he had on hand.

The liquid that was worth but one dollar, the dose being twenty drops, would be rendered of double that value by a few shakes, which would so strengthen it that ten drops would suffice! Sailors and soldiers would find this principle of great value; they would put a vial of whisky in their pockets, and, by shaking it, have grog enough for a voyage or campaign! Nay, armies might subsist on a little portable soup, increased in power and spiritualized by shaking! What an invention for starving Ireland! what a great trade shaking would be if Homœopathy were not a humbug! Instead of endeavoring to accumulate, the world would sit down satisfied to *shake* what it has already gotten?—*St. Louis Med. and Surg. Jour.* in *Western Lancet.*

